# “Why Me?!” An Attribution Perspective on Employees’ Flight-or-Fight Responses to Customer Mistreatment

**DOI:** 10.3390/bs16081385

**Published:** 2026-08-12

**Authors:** Lele Fan, Anan Fan, Lijun Chen, Wenling Shao

**Affiliations:** 1 School of Business Administration, Jimei University, Xiamen 361021, China; fanlele@jmu.edu.cn (L.F.); lijunchen@jmu.edu.cn (L.C.); swenl@126.com (W.S.); 2 Institute of Science Innovation and Culture (ISIC), Rajamangala University of Technology Krungthep (RMUTK), Bangkok 10120, Thailand

**Keywords:** experienced customer mistreatment, observed customer mistreatment, shame, hostility, psychological withdrawal behavior, service sabotage

## Abstract

Drawing on attribution theories, this study presents an integrated moderated serial mediation model to explain when and why customer mistreatment is associated with victimized employees’ contrasting “flight” and “fight” responses. Specifically, we propose that observed customer mistreatment serves as an individually perceived social cue that shapes how employees interpret and respond to their own mistreatment experiences. When similar mistreatment is rarely observed among colleagues, employees are more likely to see themselves as singled out, a perception accompanied by greater self-blame and, in turn, shame and psychological withdrawal. When such mistreatment is commonly observed, employees may instead hold customers responsible, which is linked to greater hostility and, in turn, retaliatory service sabotage. Using three-wave time-lagged data from 282 customer-service employees across various industries, we found support for the hypothesized moderating effects of observed customer mistreatment. The conditional serial indirect effect on psychological withdrawal through self-blame and shame was significant, whereas for service sabotage, the separate indirect effects through customer-directed blame and hostility were significant, but their serial indirect effect was not. These findings advance understanding of the boundary conditions and cognitive–affective processes that may underlie employees’ flight-or-fight reactions to customer mistreatment and offer implications for both theory and practice.

## 1. Introduction

Customer mistreatment refers to “the low-quality interpersonal treatment that employees receive from their customers during service interactions” (M. Wang et al., 2011). Studies across diverse service settings have documented customer mistreatment as a recurring feature of customer-facing work, encompassing unreasonable demands, abusive communication, the withholding of information needed for service delivery, and verbal or nonverbal conduct that publicly demeans employees (Chi et al., 2018; R. Shao & Skarlicki, 2014; Wu et al., 2023). Such experiences place considerable emotional strain on victimized employees and have been associated with a range of psychological costs, including intensified negative emotions (Li et al., 2021; Wong & Pan, 2023), elevated emotional exhaustion (Cheng & Jiang, 2026; Yang et al., 2020; Zhan et al., 2016), and persistent rumination (Baranik et al., 2017; M. Wang et al., 2013; H. Zhang et al., 2024). Beyond internal strain, customer mistreatment also carries behavioral consequences. Studies have shown that such experiences may be followed by destructive behaviors directed toward customers (Chi et al., 2013; Ho & Gupta, 2014; Liu et al., 2024; Walker et al., 2014; M. Wang et al., 2011; Wong & Pan, 2023), coworkers (Fan et al., 2022; Kim & Qu, 2018, 2019; Huilian et al., 2022), and family members (R. Zhang et al., 2019; Zhu et al., 2021). These behavioral consequences are also reflected in increased work withdrawal (Alola et al., 2019; Chi et al., 2018; Grandey et al., 2004; Sliter et al., 2012; X. Wang & Wang, 2017; Zhan et al., 2016), diminished organizational citizenship behavior (Amarnani et al., 2022; R. Shao & Skarlicki, 2014), and impaired service and job performance (Baranik et al., 2017; Khademi-Gerashi et al., 2024; Park & Kim, 2020).

Customer mistreatment has devastating effects on employees and their organizations, with perhaps the two most problematic consequences being that it causes employees to exhibit psychologically or physically manifested withdrawal behaviors known as “flight” responses and that it triggers them to engage in a “fight” response to retaliate against customers. Research along two lines has generally adopted a resource-based or emotion-based theoretical perspective to understand victimized employees’ unfavorable reactions. Viewed through a resource-based lens, customer mistreatment constitutes a form of resource loss because coping with excessive customer demands requires employees to draw on their limited personal resources (Yang et al., 2020). Consistent with conservation of resources theory, such loss may prompt employees to protect what remains and guard against further depletion (Hobfoll, 1989). Accordingly, victimized employees may resort to “flight” or “fight” responses to minimize the net loss of resources (Alola et al., 2019; R. Shao & Skarlicki, 2014; X. Wang & Wang, 2017; Zhan et al., 2016). In addition, emotion-based mechanisms have frequently been used to explain why customer mistreatment leads to unfavorable employee outcomes. For instance, prior studies have indicated that customer mistreatment may elicit negative emotions that increase employees’ tendency to punish the mistreating party and retaliate against perceived injustice, an explanation often grounded in negative reciprocity within social exchange theory, interpersonal justice perspectives, and affective events theory (Chi et al., 2013; Ho & Gupta, 2014; Skarlicki et al., 2008; Walker et al., 2014). Relatedly, Grandey et al. (2004), Chi et al. (2018), and Li et al. (2021) have drawn on the stressor–stress–strain logic to conceptualize customer mistreatment as a salient workplace stressor that evokes aversive emotions, including fear, irritation, and anger, thereby increasing the likelihood of withdrawal or retaliation. While these theoretical approaches may be insightful, they fail to address the puzzling question of when and why victimized employees are more likely to respond with “flight” than “fight,” or vice versa. This gap is consequential, as the relative prominence of these responses may vary across situations, despite the fact that they are not necessarily mutually exclusive and may coexist. Indeed, only by identifying the boundary conditions and psychological processes that give rise to one response over the other can scholars and managers develop more focused interventions to curtail their occurrence.

**Figure 1:**
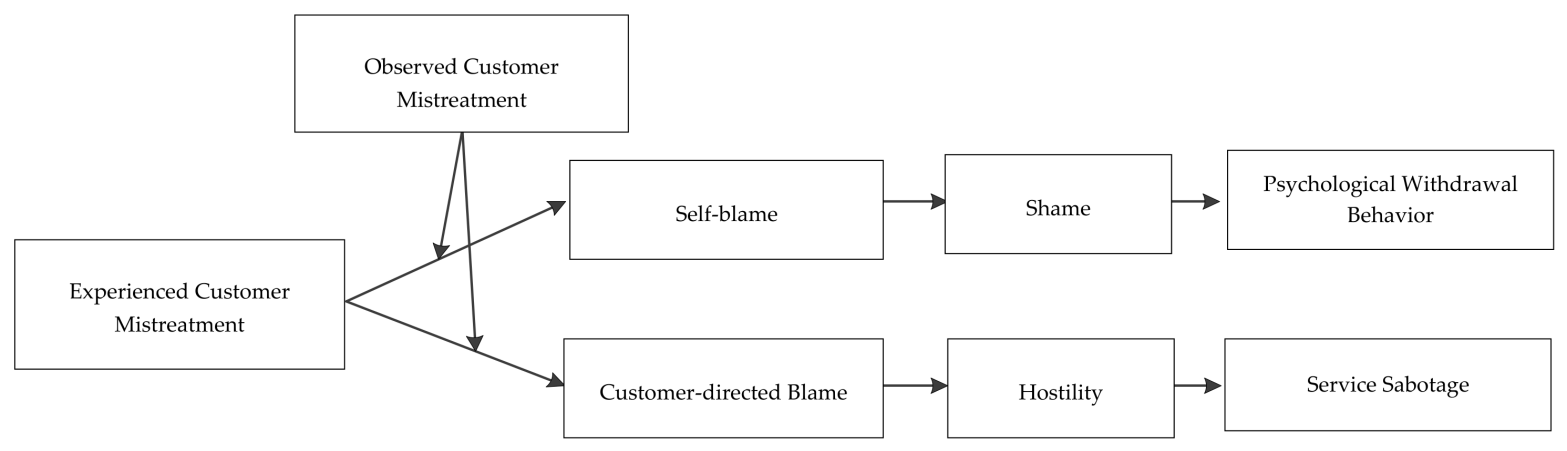
Theoretical Model.

To fill this gap, this study draws on attribution theories to examine how blame attributions shape employees’ “flight” or “fight” responses following their experiences of customer mistreatment. On the one hand, the covariation model (Kelley, 1973) helps explain why employees may be more likely to blame themselves when similar customer mistreatment is seldom observed among coworkers. Such a pattern may signal low consensus, shifting their question from “Why me?” to “Why only me?” and prompting them to locate the cause of these negative encounters within themselves. By contrast, frequent observations of similar mistreatment among coworkers may indicate high consensus, making employees more likely to attribute responsibility to customers. On the other hand, this study integrates this information-based attributional account with the belief in a just world hypothesis (BJW; Lerner & Miller, 1978) to shed light on the motivational basis of self-blame under conditions of low consensus. While consensus cues provide information for causal inference, the desire to preserve a just and orderly worldview may make employees more likely to interpret being singled out as reflecting something about themselves. Consistent with this reasoning, self-blame may represent an effort to preserve just-world beliefs and maintain a sense of control despite running counter to the need for self-improvement. Furthermore, by additionally including shame and psychological withdrawal behavior as the “flight” responses, as well as hostility and service sabotage as the “fight” responses, this study presents an integrated research model that is deeply grounded in the attribution perspective to put the diverse cognitive, emotional, and behavioral responses of victimized employees on the agenda of customer mistreatment research. The research model is illustrated in Figure 1.

The contributions of this study are threefold. First, in response to the fact that employees’ responses to their experienced mistreatment may be influenced by similar experiences they have witnessed from colleagues (e.g., Dhanani & LaPalme, 2019; Schilpzand et al., 2016; P. Shao et al., 2018; Tong et al., 2019), our research uses Kelley’s (1973) covariation model to theorize that observed customer mistreatment serves as an individually perceived social cue that may signal consensus and help explain divergent responses among victimized employees. This study suggests that when employees rarely witness similar mistreatment among colleagues, their responses are more likely to involve self-blame, shame, and psychological withdrawal. By contrast, when such mistreatment is more commonly observed among colleagues, employees are more likely to respond through customer-directed blame, hostility, and service sabotage. This distinction helps address an unresolved issue: why victimized employees may be more inclined toward a “flight” response under certain conditions and toward a “fight” response under others. Second, contributing to emerging research that views blame attribution as a socially informed judgment based on comparisons between one’s own experiences and those of others (e.g., Dhanani & LaPalme, 2019; Schilpzand et al., 2016; P. Shao et al., 2018; Tong et al., 2019), this research integrates this perspective with BJW theory to suggest that employees’ responsibility attributions are shaped by both colleagues’ mistreatment experiences as social cues and their broader beliefs about whether the world is fair. In doing so, this study extends the application of BJW as a theoretical lens for understanding employees’ self-blaming responses to customer mistreatment in organizational settings. Finally, this research uses an attribution perspective to understand employees’ flight-or-fight responses to customer mistreatment. This attribution theory approach sets this work apart from most extant studies, which have primarily explained customer mistreatment through resource loss, social exchange, justice concerns, and stress-based accounts. Such research is significant because it sheds light on the critical role that blame attribution plays in eliciting employees’ subsequent emotional and behavioral responses, thus providing additional explanations beyond resource- and emotion-based mechanisms. The literature on shame and hostility is also extended by highlighting the distinct associations between shame and employee withdrawal and between hostility and retaliation, as predicted by theoretical accounts of their social functions.

### 1.1. Theoretical Overview and Hypothesis Development

Attribution theory is not a monolithic theory; rather, it constitutes a theoretical landscape encompassing a multitude of sub-theories (attribution theories). Each of these sub-theories is dedicated to explaining specific attribution phenomena (Kelley & Michela, 1980). In particular, the covariation model, representing rational attribution theory, primarily emphasizes the use of three types of informational cues—consensus, consistency, and distinctiveness—in the attribution process to formulate logical causal inferences (Kelley, 1973). The consensus dimension of causal information assesses the extent to which mistreatment is shared among multiple individuals in a given situation. High consensus exists when the target perceives their victimization experience as widely shared, whereas low consensus occurs when the target, without other coworkers, is the sole recipient of customer mistreatment. Both the consistency and distinctiveness dimensions are based on variations within individuals, with the former assessing how consistently the target experiences mistreatment over time and the latter examining the extent to which a target faces mistreatment from multiple perpetrators rather than just one (Harvey et al., 2014). Recognizing that an employee’s response could be significantly shaped by a social referential comparison of their own experiences with those of others, rather than being solely determined by the mistreatment they personally endure from customers, our research places a specific focus on exploring the consensus dimension. Consistent with prior research showing that the frequency with which coworkers are mistreated can inform such judgments (Peng et al., 2014; Tong et al., 2019), this study treats observed customer mistreatment as a proxy for consensus information rather than as a direct measure of perceived consensus. These observations may therefore provide consensus-related cues that prompt employees to shift from the initial “Why me?” attributional question to “Am I the only one?” It is worth noting, however, that such social comparisons may occur spontaneously and outside conscious awareness (Mussweiler et al., 2004), suggesting that employees may draw on coworkers’ experiences without deliberate reflection. Accordingly, the use of consensus information in attributional judgment may not always be neutral and is often shaped by underlying motivational concerns. Yet the covariation model’s emphasis on logical causal inference leaves limited scope for considering such influences on the processing and interpretation of attributional cues. To address this limitation, our research integrates the covariation model with BJW theory to consider how employees’ motivation to preserve just-world beliefs may shape their interpretation of consensus-related cues and subsequent attributions. These attributions, in turn, influence employees’ emotional and behavioral responses. The hypotheses are elaborated in the following sections.

#### 1.1.1. Observed Customer Mistreatment as a Moderator of the Link Between Experienced Customer Mistreatment and Blame Attributions

Customer mistreatment can take the form of inappropriate gestures, offensive body language, verbal abuse, or other customer-initiated uncivil behaviors (R. Shao & Skarlicki, 2014). Despite originating outside the organization, these incidents can frustrate victimized employees’ goal pursuit and exert a substantial influence on their work experiences (M. Wang et al., 2013). When exposed to mistreatment, employees often attempt to understand its causes and who should be held responsible for it (Weiner, 1986). Given that customers are generally the source of mistreatment, employees often attribute responsibility outward to protect their self-worth and regard customers as the main party responsible for such negative experiences (i.e., self-serving bias). Consistent with this view, customer-directed blame has been examined as an important attributional response to customer mistreatment (Garcia et al., 2019). However, employees may also direct part of the blame inward and view themselves as contributing to the situation. Indeed, self-blame has been demonstrated to be a common response to victimization, illness, and physical disability in the social psychological literature (Bhuptani & Messman, 2023; Cascardi & O’Leary, 1992; Janoff-Bulman, 1979; Miller & Porter, 1983; Rancher et al., 2022; Taylor et al., 1984; Tennen et al., 1986). Although the self-blame is contrary to self-enhancement motivation, this attribution does trigger an “illusion of control” in the victim. Early research on victimization suggested that victims may take some responsibility onto themselves to make an otherwise uncontrollable event feel less arbitrary (Medea & Thompson, 1974). In addition, Kahneman and Miller (1986) made a similar point: when determining how devastating consequences might have been prevented, victims of aversive events are more likely to reflect on themselves rather than focus on other participants. More importantly, recent studies on customer mistreatment (Ni et al., 2024), as well as studies on other forms of workplace mistreatment, such as incivility (Schilpzand et al., 2016; Tong et al., 2019) and abusive supervision (Burton et al., 2014; Kedharnath et al., 2024; Pan & Sun, 2023; Tröster & Van Quaquebeke, 2021), also indicate that targets may blame themselves for their own victimization experiences.

With few exceptions (e.g., Dhanani & LaPalme, 2019; Schilpzand et al., 2016; P. Shao et al., 2018; Tong et al., 2019), blame attributions are typically investigated in a vacuum; that is, similar victimization experiences of peers and their potential impact on attributions are not taken into account. However, the covariation model posits that low consensus occurs when victims of aversive events perceive that they are being singled out. In such cases, individuals are more likely to blame themselves. Extending this reasoning to service settings, we argue that the meaning employees attach to customer mistreatment varies depending on how typical such negative experiences are within a given service organization. In particular, when hardly anyone is abused (i.e., observed customer mistreatment is low), those who are targets of customer mistreatment may transition from pondering “why me” to asking “why only me.” This shift can make it easier for them to conclude, for instance, that “I am the one who is responsible for what has happened.” Consequently, customer mistreatment can be seen as a result of personal deficiencies. Lerner and Simmons (1966), in formulating BJW theory, argued that when confronted with unfair events, individuals may interpret them through an attributional process grounded in the principle that people generally receive and deserve their outcomes. Within this framework, perceiving oneself as singled out may challenge employees’ just-world beliefs—“If the world is just and orderly, why should I, and not others, suffer this injustice?” Self-blame may therefore provide one way of relieving the resulting cognitive dissonance while preserving those beliefs. Moreover, employees’ self-protective motivation of “harm avoidance” may become more salient in such situations, potentially reinforcing the self-blaming interpretation that “something about me personally contributed to the mistreatment.” In contrast, high levels of observed customer mistreatment may signal that such experiences are widely shared, making victimized employees more likely to blame the transgressors and conclude that “it is not my fault,” a response consistent with blame avoidance and self-enhancement motives. Moving beyond prior research that has examined self-blame and customer-directed blame as separate attributional responses, our study integrates both within a unified framework and identifies observed customer mistreatment as a key boundary condition shaping their relative strength. Hence, it is hypothesized:

**H1(a).**  *The positive relationship between experienced customer mistreatment and self-blame is stronger when observed customer mistreatment is low rather than high.*

**H1(b).**  *The positive relationship between experienced customer mistreatment and customer-directed blame is stronger when observed customer mistreatment is high rather than low.*

#### 1.1.2. Blame Attributions for Customer Mistreatment and Divergent Emotional and Behavioral Responses

Previous research has shown that blame attributions for workplace mistreatment shape employees’ subsequent emotional and behavioral responses. For example, Bradfield and Aquino (1999) suggested that the blame attribution of victimized employees is significantly related to their perceptions of revenge and forgiveness. Additionally, Aquino et al. (2001) investigated the influence of blame attributions on employees’ coping strategies and found that employees’ revenge behavior increased, while their willingness to reconcile decreased when they attributed responsibility to the transgressors. More importantly, similar to research on intra-organizational abuse, such as workplace incivility and abusive supervision, victimized employees’ retaliatory reactions to customer mistreatment have been extensively validated (Chi et al., 2013; Walker et al., 2014; M. Wang et al., 2011; I. A. Wang et al., 2023; Wong & Pan, 2023). Prior studies further show that customer mistreatment can elicit “hot” emotions, such as hostility and anger, which motivate customer-directed sabotage (Chi et al., 2013; M. Wang et al., 2011; Wong & Pan, 2023). This study extends this affect-based explanation by identifying customer-directed blame as a proximal cognitive appraisal through which customer mistreatment may contribute to hostility and, ultimately, service sabotage.

Whereas external attributions direct blame toward customers, self-blame turns attention inward and typically gives rise to shame, a self-conscious emotion that reflects a negative appraisal of the self (Greenbaum et al., 2020; Ruebottom & Toubiana, 2024; Tracy & Robins, 2004). Unlike basic emotions such as hostility, shame requires self-reflection and self-evaluation (Niedenthal et al., 1994). Given the cognitive complexity of shame, Miranda et al. (2020) speculated that, instead of being directly triggered by an event itself, it is more about the pattern of cognitive appraisals made on the event. Admittedly, scholars have long described shame as an appraisal-based emotion rooted in internal attributions for negative incidents (Izard et al., 1999). While outward-directed attributions tend to elicit more basic emotional responses, internal attributions foster shame by leading victimized employees to perceive themselves as blameworthy, accountable, or responsible for what occurred. As noted above, when the observed customer mistreatment is low, victimized employees are apt to make self-blaming attributions for their own victimization experience. Those who observe less customer mistreatment around them are more likely to determine, for example, that “it’s me and I cannot do anything about it.” This self-scrutiny brings with it a painful sense of shrinking or “smallness,” as well as feelings of powerlessness and worthlessness (e.g., “I did that terrible thing, and therefore I am an incompetent, unworthy or bad person”). Thus, it is speculated that customer mistreatment would elicit a shameful response from the victimized employee, a signal that the ego is threatened and needs further protection. Shame may therefore create a desire to withdraw, as employees experiencing this emotion may feel exposed, become more concerned about others’ judgments, and seek psychological distance from the threatening situation (Daniels & Robinson, 2019; Z. Wang & Jiang, 2023). Following Lehman and Simpson (1992), withdrawal can be categorized as physical or psychological. Physical withdrawal includes observable behaviors such as absenteeism, turnover, lateness, and leaving work early, whereas psychological withdrawal involves more covert responses such as daydreaming, reduced effort, disengagement, or preoccupation with absence. While prior research has primarily examined physical withdrawal following experiences of customer mistreatment (Alola et al., 2019; Sliter et al., 2012), this study focuses on psychological withdrawal because it is subtler and better captures the defensive coping through which shamed employees protect themselves from further harm and regain a sense of security. By identifying self-blame as a cognitive antecedent of shame-related psychological withdrawal, this study extends prior research on shame and withdrawal and distinguishes this inward-directed flight response from the outward-directed fight response associated with customer-directed blame, hostility, and service sabotage. Hence, it is hypothesized:

**H2(a).**  *The positive indirect relationship between experienced customer mistreatment and psychological withdrawal via self-blame and subsequent shame is stronger when observed customer mistreatment is low rather than high.*

**H2(b).**  *The positive indirect relationship between experienced customer mistreatment and service sabotage via customer-directed blame and subsequent hostility is stronger when observed customer mistreatment is high rather than low.*

## 2. Materials and Methods

### 2.1. Sample and Procedures

Consistent with Chi et al. (2018), we recruited employees from multiple service industries across mainland China, including finance, insurance, real estate, healthcare, tourism, consulting, retail, and hospitality, to enhance the generalizability of the findings. Although these industries differed in the services they provided, all employees included in the study had regular direct contact with customers and worked in shared settings where they could observe coworkers’ interactions with customers. These work characteristics provided a common customer-facing context for examining both experienced and observed customer mistreatment and served as the basis for participant eligibility. Surveys were administered through a secure online platform. To identify potential participants, we used the contact information available in an alumni directory to reach alumni who were currently employed. During the initial contact, we introduced the study and explained the eligibility criteria. Alumni who met the criteria were invited to participate, and those contacted were also asked to refer coworkers or other employees who satisfied the same requirements. We then contacted the referred individuals directly, confirmed their eligibility, and provided the survey link and study instructions to those who agreed to participate. Incentives were provided for both survey participation and recruitment assistance. Similar recruitment procedures have been used in prior organizational research (e.g., Greenbaum et al., 2012, 2014; Jalil et al., 2022; Mitchell et al., 2015). This procedure enabled us to recruit employees across multiple service industries while reducing reliance on any single organization or occupational group.

To mitigate common method bias (CMB), the survey was administered at three time points separated by one-month intervals (Podsakoff et al., 2003). To match responses across waves while preserving anonymity, participants provided a four-digit identification code based on the last four digits of a telephone number. We also incorporated a critical incident recall procedure into the survey design (Flanagan, 1954), an approach that has been shown to be effective in examining individuals’ perceptions of and responses to workplace mistreatment (e.g., Bedi & Schat, 2017; Fan et al., 2026; Hershcovis et al., 2017, 2018; Mitchell et al., 2015). At Time 1 (T1), before completing the focal measures, participants were asked to recall and briefly describe specific incidents of customer mistreatment that they had personally experienced and incidents that they had observed coworkers experiencing during the preceding six months. This procedure was intended to facilitate more vivid and specific recall. Participants then completed measures of experienced and observed customer mistreatment, social desirability bias, and demographic characteristics. Of the 547 questionnaires initially distributed, 391 usable responses were received, corresponding to a response rate of 71.48%. One month later, at Time 2 (T2), valid T1 respondents were instructed to recall both types of incidents identified at T1 before reporting self-blame, customer-directed blame, shame, and hostility. Among the 391 participants contacted at this stage, 327 provided valid responses, corresponding to a response rate of 83.63%. At Time 3 (T3), conducted one month after T2, the 327 valid T2 respondents were again instructed to recall the personally experienced incidents identified at T1 before reporting psychological withdrawal and service sabotage. At this final wave, 293 valid responses were retained, corresponding to a response rate of 89.60%.

We implemented several procedures to protect participants and maintain survey quality. First, before completing each online questionnaire, participants were informed that participation was voluntary and that they could withdraw at any time without penalty. The study introduction emphasized the importance of providing honest and conscientious responses. Participants were also assured that their data would remain confidential and would be used solely for research purposes. Second, the IP addresses and timestamps were recorded when the participants completed the online questionnaires. These data were then checked to ensure that each participant completed the questionnaires at three time points and that the IP addresses of the questionnaires submitted by different participants were different. After eliminating invalid questionnaires due to data matching failure, 282 valid questionnaires were obtained. To assess potential attrition bias, we compared these 282 retained participants with the 109 T1 respondents not included in the final sample across all available T1 variables. Independent-samples *t*-tests indicated no significant differences between retained and nonretained participants in experienced customer mistreatment, t (389) = −1.21, *p* = 0.23, observed customer mistreatment, t (389) = −1.34, *p* = 0.18, or social desirability, Welch’s t (328.13) = −0.66, *p* = 0.51. Chi-square tests also indicated no significant differences in gender, χ^2^ (1, *N* = 391) = 0.30, *p* = 0.58, or education, χ^2^ (3, *N* = 391) = 4.15, *p* = 0.25. However, significant differences were found in age category, χ^2^ (4, *N* = 391) = 50.85, *p* < 0.01, and tenure category, χ^2^ (4, *N* = 391) = 61.38, *p* < 0.01. These findings indicate that nonretention was not systematically associated with the focal T1 variables or social desirability, although some demographic selectivity related to age and tenure cannot be ruled out. The final sample comprised 53.55% male and 46.45% female participants. They worked in a variety of occupations, including finance and insurance (25.53%), real estate (17.02%), healthcare (19.15%), tourism (13.83%), consulting (9.57%), and others (14.89%). Regarding age, 31.21% of participants were 25 years old or younger, 37.23% were 26 to 35 years old, 21.99% were 36 to 45 years old, and 9.58% were 46 years old or older. Regarding education, 19.50% had completed junior or high school, 30.14% held a junior college degree, 37.23% had a bachelor’s degree, and 13.12% had a master’s degree or higher. In terms of work experience, 41.84% had worked for less than one year, 24.11% for one to three years, 17.38% for three to five years, 7.80% for five to ten years, and 8.87% for more than ten years.

### 2.2. Variables and Measurements

Before data collection, all scale items were translated and back-translated according to Brislin’s (1980) procedure to ensure semantic equivalence. Unless otherwise noted, items were rated on a seven-point Likert-type scale ranging from 1 = “strongly disagree” to 7 = “strongly agree.”

### 2.3. Experienced Customer Mistreatment

Experienced customer mistreatment was assessed with R. Shao and Skarlicki’s (2014) five-item scale. The measure was well suited to our study because its items were derived from critical incidents in face-to-face service encounters. Participants indicated how frequently they had personally experienced each negative customer behavior during the previous six months (α = 0.89). Sample items included “Criticized you in front of your coworkers or supervisors” and “Yelled at you.”

### 2.4. Observed Customer Mistreatment

Observed customer mistreatment was assessed with R. Shao and Skarlicki’s (2014) scale, with the wording revised to shift the referent from employees’ own experiences to their observations of coworkers’ experiences. Participants rated how often they had witnessed customers mistreating their coworkers during the previous six months (α = 0.90). Sample items included “Criticized coworkers in front of you or your supervisors” and “Yelled at your coworkers”.

### 2.5. Self-Blame

Self-blame was assessed with four items adapted from Garnefski et al. (2001). Respondents rated items capturing the degree to which they held themselves responsible for customers’ negative behaviors (α = 0.87). Sample items included “I felt that I am the one to blame for the customers’ negative behaviors” and “I think that basically the cause for negative interactions must lie within myself.”

### 2.6. Customer-Directed Blame

Using Garnefski et al.’s (2001) four-item subscale of blaming others, customer-directed blame was measured. Sample items included “I felt that customers are responsible for what has happened” and “I felt that the cause lies with customers” (α = 0.88). The scale was slightly modified by replacing “others” with “customers,” and participants rated the degree to which they held customers responsible for the mistreatment they had experienced.

### 2.7. Shame

Shame was assessed using five items from Marschall et al.’s (1994) State Shame and Guilt Scale (SSGS). Sample items included “I feel worthless, powerless” and “I want to sink into the floor and disappear.” Participants reported the degree to which each item captured their feelings in response to customers’ negative behaviors (α = 0.95).

### 2.8. Hostility

Hostility was assessed with five items from Watson and Clark’s (1994) Positive and Negative Affect Schedule, Expanded Form (PANAS-X). Sample items for hostility include “irritable,” “disgusted,” and “scornful.” Participants indicated the extent to which each item reflected their feelings in response to customers’ negative behaviors (α = 0.94).

### 2.9. Psychological Withdrawal Behaviors

We assessed psychological withdrawal behaviors using Lehman and Simpson’s (1992) eight-item scale. Sample items included “Thoughts of being absent” and “Let others do your work.” Participants rated the extent to which they had engaged in these behaviors (α = 0.95).

### 2.10. Service Sabotage

Service sabotage was assessed using Chi et al.’s (2015) six-item scale designed for face-to-face service settings. Sample items included “Intentionally hurrying customers when you want to” and “Behaving negatively toward customers.” Participants rated the extent to which they had engaged in these behaviors (α = 0.94).

### 2.11. Control Variable

Demographic characteristics, including gender, age, education, and tenure, have been associated with withdrawal behavior (Blau, 1985) and were therefore controlled to rule out alternative explanations. Additionally, social desirability bias was included as a covariate to control for its potential adverse impact on the research results. Social desirability bias was assessed with Winkler et al.’s (2006) six-item short form of the Balanced Inventory of Desirable Responding (BIDR), which was later used by Goetzke et al. (2014). The scale showed acceptable reliability (α = 0.72).

### 2.12. Ethics Approval and Data Protection

This study was conducted with the approval of the Ethics Review Board of Jimei University (Approval No.: 20250613921). Participants were informed of the study purpose, voluntary participation, confidentiality, and the recording of IP addresses and timestamps for response matching and data-quality screening, and all provided informed consent. A four-digit code based on the last four digits of a telephone number was used to match responses across waves; full telephone numbers and other direct identifiers were not collected. IP addresses and timestamps were stored in password-protected files accessible only to the research team and deleted after screening and matching.

## 3. Results

### 3.1. Confirmatory Factor Analysis

Before examining the hypotheses, we used Mplus 7.4 to conduct confirmatory factor analyses (CFAs) with maximum-likelihood (ML) estimation and evaluate the convergent validity of the study constructs (Muthén & Muthén, 1998–2012). The hypothesized model contained eight factors: experienced customer mistreatment, observed customer mistreatment, self-blame, customer-directed blame, shame, hostility, psychological withdrawal behaviors, and service sabotage. The results indicated that the eight-factor model fit the data (χ^2^ = 1570.22, df = 791, CFI = 0.92, TLI = 0.91, RMSEA = 0.06, SRMR = 0.05). Furthermore, the model comparisons reported in Table 1 indicated that the eight-factor model fit the data significantly better than the alternative specifications, supporting the discriminant validity of the measures. The chi-square difference test further indicated that the eight-factor model fit the data significantly better than the one-factor model (∆χ^2^ = 6762.12; ∆df = 28; *p* < 0.01), suggesting that CMB was unlikely to be a serious concern in this study.

### 3.2. Descriptive Statistics and Correlation

Table 2 summarizes the descriptive statistics, zero-order correlations, and reliability estimates for the study variables.

### 3.3. Hypothesis Test Results

**Figure 2:**
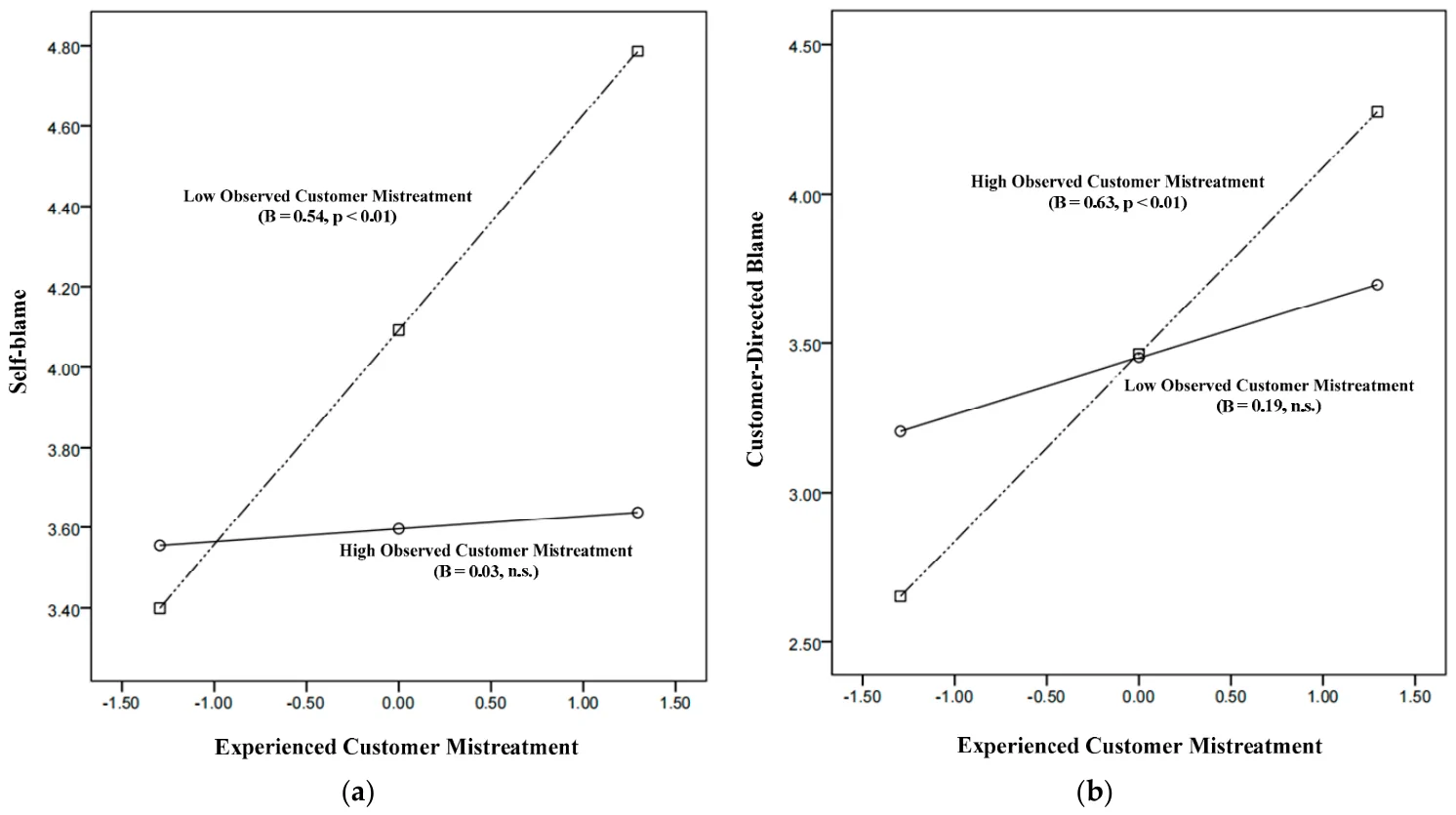
(**a**) Moderating Effect of Observed Customer Mistreatment on the Relationship Between Experienced Customer Mistreatment and Self-blame; (**b**) Moderating Effect of Observed Customer Mistreatment on the Relationship Between Experienced Customer Mistreatment and Customer-Directed Blame.

**Figure 3:**
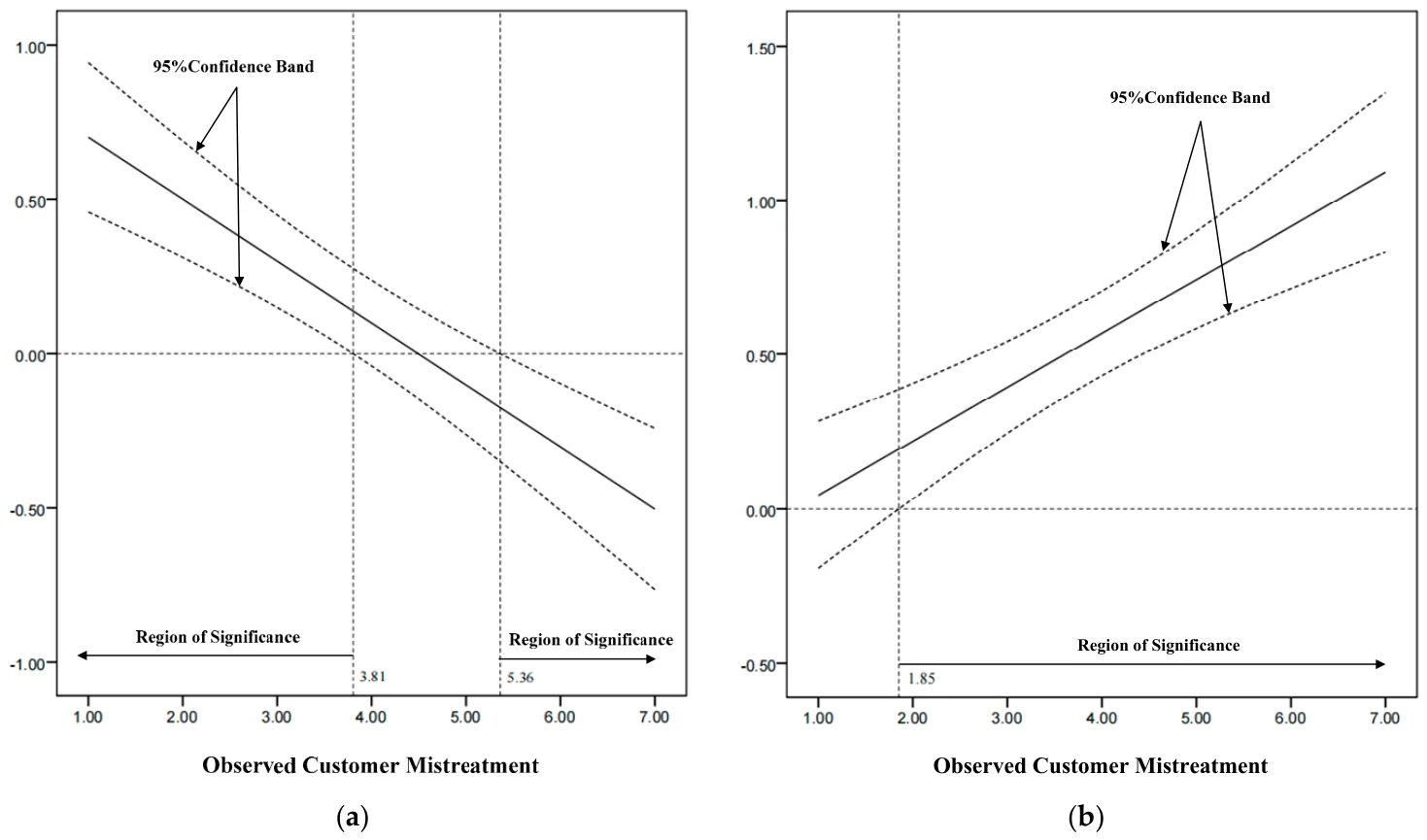
(**a**) Conditional Effect of Experienced Customer Mistreatment on Self-blame at Values of Observed Customer Mistreatment; (**b**) Conditional Effect of Experienced Customer Mistreatment on Customer-Directed Blame at Values of Observed Customer Mistreatment. Note. The solid line represents the point estimate, while the two diagonal dashed lines indicate the 95% confidence band.

We examined the research model and proposed hypotheses using Model 83 in PROCESS v3.2 (Hayes, 2018). PROCESS Model 83 was selected primarily because its specification corresponds to the hypothesized moderated serial mediation structure and allows direct estimation of the conditional indirect effects and the index of moderated mediation using bootstrap confidence intervals. It also provides an accessible and user-friendly procedure, and recent studies have similarly employed Model 83 to test comparable moderated serial mediation models (Marr et al., 2025; Paustian-Underdahl et al., 2025). The significance of the indirect effects was assessed through 10,000 bootstrap resamples, with bias-corrected 95% confidence intervals used for inference (MacKinnon et al., 2004). H1(a) and H1(b) predicted that observed customer mistreatment would moderate the association between experienced customer mistreatment and blame attributions. To test these interaction effects, the two mistreatment variables were mean-centered before the interaction term was created (Dawson, 2014). As presented in Table 3a,b, the interaction term was negatively correlated with self-blame (B = −0.20, *p* < 0.01) and positively correlated with customer-directed blame (B = 0.17, *p* < 0.01). Simple slope tests further showed that experienced customer mistreatment positively predicted self-blame when observed customer mistreatment was low (B = 0.54, *p* < 0.01), but the effect was nonsignificant when the moderator was high (B = 0.03, n.s.), supporting Hypothesis 1(a) (Aiken et al., 1991; see Figure 2a). By contrast, experienced customer mistreatment positively predicted customer-directed blame when observed customer mistreatment was high (B = 0.63, *p* < 0.01), but not when the moderator was low (B = 0.19, n.s.), supporting Hypothesis 1(b) (see Figure 2b). The Johnson-Neyman technique was then used to identify the regions of observed customer mistreatment in which the conditional effects of experienced customer mistreatment on blame attributions were significant. As shown in Figure 3a, experienced customer mistreatment was positively associated with self-blame when observed customer mistreatment was below 3.81 on the 7-point scale. This effect was nonsignificant between 3.81 and 5.36, but became significantly negative when observed customer mistreatment exceeded 5.36. On the contrary, as shown in Figure 3b, as long as the value of observed customer mistreatment was higher than 1.85, experienced customer mistreatment was significantly positively correlated with customer-directed blame. This pattern points to a more nuanced conclusion: Self-blame was more likely to appear at lower levels of observed customer mistreatment, whereas customer-directed blame became more prominent at higher levels. However, the two attributional responses were not mutually exclusive. Within the 1.85 to 3.81 range of observed mistreatment, increases in experienced customer mistreatment were accompanied by higher levels of both self-blame and customer-directed blame, suggesting that employees may assign responsibility to both themselves and customers.

H2(a) proposed that experienced customer mistreatment would exert a serial indirect effect on psychological withdrawal behaviors through self-blame and shame, and that this effect would be amplified at lower levels of observed customer mistreatment. In the first part of Table 4a, the conditional indirect effect through self-blame and shame was significant at lower observed customer mistreatment (effect = 0.041, bootstrap 95% CI [0.014, 0.077]), but nonsignificant at higher observed customer mistreatment (effect = 0.003, bootstrap 95% CI [−0.010, 0.016]). Additionally, the confidence interval for the index of moderated mediation excluded zero (Index = −0.016, bootstrap 95% CI [−0.027, −0.005]), providing support for H2(a). The PROCESS Model 83 results further indicated that self-blame also served as a separate mediator in the conditional indirect association between experienced customer mistreatment and psychological withdrawal behaviors. As presented in the middle part of Table 4a, the self-blame pathway yielded a significant conditional indirect effect at low observed customer mistreatment (effect = 0.075, bootstrap 95% CI [0.004, 0.170]), whereas the corresponding effect at high observed customer mistreatment was not significant (effect = 0.004, bootstrap 95% CI [−0.019, 0.033]). More convincingly, the moderated mediation index was significantly lower than zero (index = −0.028, bootstrap 95% CI [−0.063, −0.001]). Moreover, the results in the last part of Table 4a indicated that experienced customer mistreatment had a significant positive indirect effect on employees’ psychological withdrawal behaviors through shame alone (effect = 0.039, bootstrap 95% CI [0.008, 0.087]).

Finally, we used the same analytical procedure to examine H2(b), which proposed that experienced customer mistreatment would predict service sabotage indirectly through customer-directed blame and hostility, with this effect expected to be stronger under higher observed customer mistreatment. The first part of Table 4b indicated that the indirect effect through customer-directed blame and hostility was nonsignificant at both high observed customer mistreatment (effect = 0.029, bootstrap 95% CI [−0.001, 0.064]) and low observed customer mistreatment (effect = 0.009, bootstrap 95% CI [−0.001, 0.028]). Furthermore, the confidence interval of the moderated mediation index also contained zero (Index = 0.008, bootstrap 95% CI [−0.0004, 0.0177]). Thus, the findings failed to support Hypothesis 2(b). However, the results presented in the middle part of Table 4b revealed a significant indirect effect of experienced customer mistreatment on service sabotage through customer-directed blame at high observed customer mistreatment (effect = 0.088, bootstrap 95% CI [0.019, 0.170]), whereas this effect was not significant at low observed customer mistreatment (effect = 0.027, bootstrap 95% CI [−0.004, 0.071]). More convincingly, the moderated mediation index was significantly greater than zero (Index = 0.025, bootstrap 95% CI [0.005, 0.051]). In addition, the results in the last part of Table 4b showed a significant indirect effect of experienced customer mistreatment on service sabotage through hostility alone (effect = 0.099, bootstrap 95% CI [0.042, 0.161]). Thus, although the serial mediation path through customer-directed blame and hostility was not significant, experienced customer mistreatment still contributed to service sabotage through two separate routes, one based on customer-directed blame and the other on hostility. In this sense, service sabotage may take either a conscious and deliberate form associated with thoughts of revenge or a more impulsive form associated with hostility, without implying that the two necessarily unfold in sequence.

## 4. Discussion

Our research developed and tested an attribution-based moderated serial mediation model to explain why employees may respond to customer mistreatment through either “flight” or “fight.” Specifically, experienced customer mistreatment showed a stronger positive relation with self-blame when observed customer mistreatment was low, but a stronger positive relation with customer-directed blame when observed customer mistreatment was high, supporting H1(a) and H1(b). These findings are consistent with the covariation model and extend prior research showing that targets may engage in self-blame across different forms of workplace mistreatment (Burton et al., 2014; Kedharnath et al., 2024; Ni et al., 2024; Pan & Sun, 2023; Schilpzand et al., 2016; Tong et al., 2019; Tröster & Van Quaquebeke, 2021). Self-blame, in turn, showed a significant indirect association with psychological withdrawal through shame. The conditional serial indirect effect was significant when observed customer mistreatment was low, supporting H2(a). This finding indicates that employees who perceive themselves as distinct targets are more likely to turn blame inward and report higher levels of shame and psychological withdrawal. By examining this conditional serial indirect effect, our study extends prior theorising and empirical evidence on the links between self-directed attributions and shame and between shame and withdrawal (Daniels & Robinson, 2019; Tracy & Robins, 2004; Z. Wang & Jiang, 2023).

By contrast, H2(b) was not supported because the conditional serial indirect effect through customer-directed blame and hostility was not significant. Nevertheless, experienced customer mistreatment had a significant indirect effect on service sabotage through customer-directed blame when observed customer mistreatment was high, as well as a significant indirect effect through hostility. One possible explanation is that customer-directed blame does not necessarily translate into hostility. Although holding the customer responsible may direct employees’ negative evaluations toward the customer, this attribution may also help protect their self-esteem by making them less likely to interpret the mistreatment as evidence of personal inadequacy. The resulting reduction in self-threat may weaken shame and may even be accompanied by relief, a pleasant emotional experience associated with successful threat avoidance (Leng et al., 2024). Relief may coexist with hostility, making employees’ emotional responses less uniformly negative and potentially preventing hostile feelings from escalating, particularly when organizational emotional display rules encourage restraint and employees exercise greater emotional self-control. Customer-directed blame may nevertheless promote service sabotage through an alternative cognitive pathway. By identifying the customer as responsible, employees may perceive retaliation against the customer as more justified, allowing service sabotage to occur without hostility being required as an emotional mediator (Aquino et al., 2001; Bradfield & Aquino, 1999). Hostility may instead arise more directly from the aversive encounter and independently contribute to service sabotage (Chi et al., 2013; M. Wang et al., 2011; Wong & Pan, 2023). Thus, the “flight” response was consistent with the proposed attribution–emotion–behavior sequence, whereas the “fight” response appeared to involve partly distinct attributional and affective routes. This asymmetry provides a more nuanced basis for the theoretical and practical implications discussed below.

### 4.1. Theoretical Implications

This study has important theoretical implications. First, the integration of the covariation model and BJW theory enables us to develop a conceptual model driven more by theory than by phenomena. Although customer mistreatment research has advanced across different streams, a unified framework is still needed to explain why victimized employees sometimes withdraw from the situation yet retaliate in other cases. This theoretical integration allows us to position observed mistreatment among colleagues as a boundary condition that helps explain the relative prominence of self-blame and customer-directed blame, with implications for employees’ subsequent responses to their own mistreatment. Our empirical results support that an employee’s response is based not so much on the mistreatment of customers they have experienced but rather on a social referential comparison of their own experience with that of others. When employees perceive their victimization as an experience unique to them, they may internalize responsibility for the mistreatment, experience shame, and ultimately withdraw psychologically from work. Conversely, customer-directed blame and hostility may each contribute to service sabotage. To be specific, employees who blame customers may perceive retaliation as justified and engage in service sabotage in an attempt to get even. This finding provides a framework for reconciling the two lines of research on employee withdrawal and retaliation, thus answering the question of when and why employees are more likely to “flight” than “fight” and vice versa.

Second, this study extends the literature on self-blame by investigating this seemingly counterintuitive attribution among employees who experience customer mistreatment. To date, most self-blame research has focused on relevant topics in the field of social psychology, such as sexual violence (Fetchenhauer et al., 2005; Janoff-Bulman, 1979), battery (Cascardi & O’Leary, 1992; Miller & Porter, 1983), and disease (Taylor et al., 1984; Tennen et al., 1986). Organizational scholars have only recently begun to examine whether victimized employees may attribute responsibility for workplace mistreatment to themselves, with existing work focusing mainly on incivility (Schilpzand et al., 2016; Tong et al., 2019) and abusive supervision (Burton et al., 2014; Tröster & Van Quaquebeke, 2021). Despite this emerging interest, BJW theory has rarely been applied to interpret victims’ self-blame, as it has traditionally been used primarily to explain why third-party observers blame victims to preserve a view of the world as just and controllable (Fan et al., 2025; Skarlicki et al., 1998; Skarlicki & Kulik, 2005; Skarlicki & Turner, 2014). By shifting the focus from observers’ victim blaming to victims’ self-blame, our study suggests that BJW theory may also provide a useful lens for understanding why employees blame themselves for customer mistreatment despite the common-sense logic of self-enhancement. Viewed through this lens, self-blame may be interpreted as a defensive attribution associated with construing adverse events as controllable and future harm as avoidable (Lerner & Miller, 1978; Shaver, 1970). This account also echoes Shaver’s (1970) argument that self-blame may serve as an instinctive defensive response through which individuals attempt to “avoid harm” when confronted with threatening events. Our study therefore offers one possible explanation for why blaming oneself rather than others is a common victim response to negative events, as documented in both organizational behavior and psychological research (Cascardi & O’Leary, 1992; Miller & Porter, 1983; Schilpzand et al., 2016; Taylor et al., 1984; Tennen et al., 1986; Tong et al., 2019; Tröster & Van Quaquebeke, 2021).

Finally, by integrating attribution theories with customer mistreatment literature, a novel perspective was developed to understand employee fight-or-flight responses to customer mistreatment. This attribution perspective on adverse employee reactions differs from previous theoretical perspectives. For example, prior studies have applied conservation of resources theory, affective events theory, social exchange theory, equity and justice perspectives, and the stressor–stress–strain framework to account for employee withdrawal and retaliation. These explanations generally portray such behaviors either as discretionary efforts to protect valued resources or as affectively charged reactions to threatening workplace events (Chi et al., 2013; Ho & Gupta, 2014; Walker et al., 2014; X. Wang & Wang, 2017). However, it is assumed that psychological withdrawal behavior and service sabotage may also result from self- and customer-directed blame attributions and the resulting emotions, respectively. This study contributes by highlighting the role of blame attributions in understanding employees’ divergent “flight” and “fight” responses in the context of customer mistreatment, thereby extending existing resource- and emotion-based explanations. Notably, this research does not discount the role of emotions in shaping employee behavior. Rather, it advances this line of inquiry by showing that shame and hostility help account for these distinct behavioral outcomes, with shame linked to psychological withdrawal and hostility to retaliation. In sum, this study extends customer mistreatment research by clarifying the moderating role of observed mistreatment and delineating distinct cognitive–affective pathways from employees’ own mistreatment experiences to psychological withdrawal and service sabotage.

### 4.2. Practical Implications

This study is also of practical importance. First, managers should acknowledge that employees’ responses to customer mistreatment are not formed in isolation, as employees may experience such mistreatment themselves while also observing colleagues undergo similar negative encounters, and these observations can influence how they interpret and respond to such mistreatment (Skarlicki & Kulik, 2005). The influence of such observations may be particularly consequential in the customer-contact service sectors represented in this study, where recurring problems involving prices or fees, claims, contracts, waiting times, booking difficulties, product availability, or service delays may subject different employees to similar negative reactions from customers. These shared conditions render coworkers’ experiences an important attributional cue for judging whether responsibility for the mistreatment lies primarily with themselves or with customers. The findings suggest that when employees rarely observe similar mistreatment among coworkers, they may be more likely to see themselves as distinct targets and to report higher levels of self-blame, shame, and psychological withdrawal from work. Managers should therefore consider that self-blame may offer employees a way of construing the experience as more controllable and future harm as more avoidable. Under such circumstances, shame may reflect the self-threatening nature of the experience, whereas psychological withdrawal may represent an effort to protect threatened self-esteem and self-worth from further harm. In this sense, this form of withdrawal should not necessarily be treated as behavior intended to harm the organization; rather, it may be better understood as a passive and avoidant form of self-protection. In contrast, service sabotage represents a more deliberate and potentially harmful employee response that may become more likely when employees perceive similar mistreatment among coworkers and attribute responsibility to customers, thereby warranting closer managerial monitoring and preventive intervention. Given that these two behavioral responses of victimized employees have significantly different negative effects on the organization, managers should determine which response is more likely to occur by assessing the extent to which employees perceive their victimization experiences as isolated or shared by coworkers, making it possible to curb their occurrence in a targeted way.

Second, this study offers attribution-based guidance for organizational intervention. Managers are encouraged to help employees reconsider their blame attributions and manage the emotions arising from customer mistreatment, thereby strengthening their capacity to restrain unfavorable behaviors when these encounters cannot be fully avoided or controlled. More specifically, for employees who see themselves as distinct targets, managers should, on the one hand, help them recognize that just-world beliefs may contribute to unwarranted self-blame and reassure them that the mistreatment does not necessarily reflect deficiencies in their abilities, qualities, or other personal characteristics. This reassurance should be supported by a timely and nonpunitive debriefing that distinguishes responsibility attributable to employees’ own actions from that arising from organizational policies, service procedures, or operational constraints beyond their control. On the other hand, managers should provide training in the service skills required in customer-contact service jobs, including responding to unreasonable customer demands, managing rude, accusatory, or hostile behavior, and redirecting escalating interactions toward constructive problem resolution, thereby strengthening employees’ service competence as well as their self-esteem and self-worth. By contrast, for employees who perceive their victimization experiences as widely shared by others, managers should acknowledge customers’ responsibility for inappropriate conduct while helping employees consider the broader service context in which such incidents occur, thereby preventing customer-directed blame from developing into revenge-oriented thoughts. Clear escalation and handover procedures should enable employees to seek managerial intervention or transfer difficult interactions when customer mistreatment persists. Regular training in emotion regulation and de-escalation may further help employees control “hot” emotions such as hostility and reduce the likelihood that these emotions culminate in service sabotage.

### 4.3. Limitations and Future Research Directions

Despite these contributions, several limitations should be noted. First, this study incorporated both employee self-blame and customer-directed blame into the research model and found that one form of blame tended to dominate when employees perceived their victimization as either relatively isolated or widely shared. It is worth noting, however, that these two seemingly contradictory attributions may sometimes coexist, with employees attributing responsibility for the same mistreatment episode to both themselves and customers. The two attributions may also vary in prominence over time. Employees may experience shame and hostility in parallel, potentially alongside relief, while the corresponding behavioral tendencies toward psychological withdrawal and service sabotage may likewise overlap or unfold sequentially. Thus, future research should further examine how the interplay between these blame attributions unfolds over time and relates to employees’ emotional and behavioral responses. This line of inquiry could also move beyond self- and customer-directed blame by considering whether victimized employees assign responsibility to the organization or to family members. As prior studies have shown, customer mistreatment may trigger displaced deviance toward workplace targets as well as aggression displaced onto family members, allowing its effects to spill over across work and family domains (Fan et al., 2022; Zhu et al., 2021). Thus, this study indicates that future research can apply this novel perspective to explore other possible attributions of blame by employees, thereby proposing a cognition-based mechanism that can be used to explain the “spillover” effects of customer mistreatment.

Second, although this study considered employee flight-or-fight responses to customer mistreatment, future research should investigate other possible responses. In line with most other research on customer mistreatment, this study failed to break through the dominant negative-reciprocity logic underlying employee-customer interactions. Studies have begun to suggest, however, that in certain situations, workplace mistreatment can lead victimized employees, driven by guilt or a willingness to reconcile, to help transgressors. Unfortunately, this line of inquiry is still in its infancy and has mainly focused on two types of interpersonal mistreatment: abusive supervision (Andiappan & Treviño, 2010; Mitchell & Ambrose, 2012; Tong et al., 2019; Tröster & Van Quaquebeke, 2021) and workplace incivility (Tong et al., 2019). Thus, little is known about whether victimized employees respond constructively to customer mistreatment, which has a direct bearing on customer satisfaction. Therefore, future research is warranted to examine this possibility among employees exposed to customer mistreatment, thereby broadening understanding of the consequences of such mistreatment. The multi-industry composition of the sample also warrants consideration. Although the sampled industries differ in service content, employees across these industries share a comparable work context in which they interact directly with customers and coworkers’ experiences can serve as consensus cues when interpreting their own mistreatment. This common basis allows the attributional processes underlying employees’ flight-or-fight responses to be examined beyond any single industry. Nevertheless, this study did not examine whether the proposed relationships vary across industries or, if so, which contextual characteristics may account for such variation. Future research should address this issue through direct cross-industry comparisons. Given cultural differences between Eastern and Western settings, extending the present findings to Western contexts also requires caution (R. Shao & Skarlicki, 2014; Zhu et al., 2021). In collectivist cultures, employees may place greater emphasis on fulfilling their role obligations and therefore interpret customer mistreatment as reflecting their own shortcomings, thereby increasing self-blame and intensifying shame. Concerns for relational harmony may further lead employees to view their relationship with customers as requiring greater restraint, with withdrawal and covert service sabotage representing less confrontational responses. By contrast, employees in individualist cultures, which prioritize autonomy, personal boundaries, and direct expression, may be less likely to internalize responsibility and more likely to attribute blame to the customer, experience hostility, and engage in more overt retaliation. Future studies should therefore examine this research model with Western samples to assess its cross-cultural generalizability.

Finally, several limitations related to measurement, study design, and the analytical approach should be acknowledged. One concern is that respondents’ level of BJW was not directly measured in this study. Although BJW theory provides a plausible explanation for why employees may engage in self-blame, the motivation to preserve just-world beliefs was not empirically tested. It therefore remains unclear whether, and to what extent, this motivation shapes employees’ attributional responses. Future research should incorporate validated measures of BJW and examine its mediating or moderating role in employees’ responses to customer mistreatment. Another concern stems from the study’s single-source design, as all measures relied on reports from victimized employees. It should be noted, however, that self-blame, customer-directed blame, shame, hostility, and social desirability bias as a control variable were appropriately assessed through self-report measures, as these constructs reflect subjective appraisals and affective states that are not readily observable by others. Additionally, self-report measures are appropriate for assessing psychological withdrawal and service sabotage, given that these behaviors are often covert and difficult for external observers to detect. Berry et al. (2012) further suggested that self-report measures may be especially useful for assessing sensitive variables, as they can yield more accurate information than other reporting sources. Given that data collection necessarily relied on a single source, two procedures were implemented to mitigate potential CMB. The first involved explaining the purpose of the study to respondents and assuring them that their responses would remain confidential. The second involved measuring the study variables at three time points, spaced one month apart, thereby temporally separating the measurement occasions in accordance with the proposed theoretical sequence (Podsakoff et al., 2012).

Despite these procedures, the time-lagged design, which relied exclusively on self-reported data, limits both causal inference and the assessment of within-person dynamics. Because the design does not establish the precise timing of the attributional, emotional, and behavioral responses relative to the mistreatment incidents, the temporal ordering of the focal variables cannot be determined conclusively, and alternative or reciprocal relationships remain possible. This concern is particularly relevant to the proposed sequence from self-blame to shame. Although Tröster and Van Quaquebeke (2021) examined guilt rather than shame, their conceptualization of self-blame as preceding a self-conscious emotional response provides indirect support for this sequence. Nevertheless, the research design does not permit a definitive test of this ordering or rule out the possibility that shame precedes or shapes self-blame. Nor can it capture how the relative prominence of self-blame and customer-directed blame varies over time or how these dynamics relate to employees’ emotional and behavioral responses. Future research could therefore use experience sampling designs to better assess temporal ordering and examine these within-person dynamics. A further concern relates to the operationalization of consensus, as our measure of observed customer mistreatment captured only the frequency with which employees observed coworkers being mistreated and functioned only as a proxy for consensus information. It thus neither directly assessed perceived consensus nor allowed us to determine whether employees used coworkers’ mistreatment as a reference point when making sense of their own experiences. Given that attributional judgments may arise without conscious deliberation, employees may not even realize that they are making such comparisons, making perceived consensus difficult to capture fully through retrospective self-report. To address this limitation, future studies could use experimental designs to manipulate consensus levels and include a post-manipulation measure of perceived consensus, thereby enabling stronger causal inferences about its effects on employees’ attributions and subsequent emotional and behavioral responses. A final analytical limitation concerns the use of PROCESS Model 83 with observed composite scores. Although Model 83 was well suited to the hypothesized conditional serial mediation structure, the analysis did not account for measurement error in the structural paths. Future research could test the full model using latent-variable SEM to provide a more stringent assessment of the hypothesized relationships.

## Figures and Tables

**Table 1:** Results of CFAs.

Model	χ^2^	df	CFI	TLI	RMSEA	SRMR
8-factor model	1570.22	791	0.92	0.91	0.06	0.05
7-factor model (1)	1957.55	798	0.88	0.87	0.07	0.06
7-factor model (2)	2047.49	798	0.87	0.86	0.08	0.06
7-factor model (3)	2160.74	798	0.86	0.85	0.08	0.08
6-factor model (1)	3189.41	804	0.76	0.74	0.10	0.10
6-factor model (2)	3461.16	804	0.73	0.71	0.11	0.10
5-factor model	3944.37	809	0.68	0.66	0.12	0.15
4-factor model	5120.18	813	0.56	0.53	0.14	0.15
3-factor model	5429.03	816	0.53	0.50	0.14	0.15
2-factor model	7024.73	818	0.37	0.33	0.16	0.19
1-factor model	8332.34	819	0.23	0.19	0.18	0.19

Note. *N* = 282; 7-factor model (1): experienced customer mistreatment + observed customer mistreatment; 7-factor model (2): self-blame + shame; 7-factor model (3): customer-directed blame + hostility; 6-factor model (1): customer-directed blame + hostility + service sabotage; 6-factor model (2): self-blame + shame + psychological withdrawal behavior; 5-factor model: self-blame + customer-directed blame + shame + hostility; 4-factor model: experienced customer mistreatment + observed customer mistreatment, self-blame + customer-directed blame, shame + psychological withdrawal behavior, hostility + service sabotage; 3-factor model: experienced customer mistreatment + observed customer mistreatment, self-blame + shame + psychological withdrawal behavior, customer-directed blame + hostility + service sabotage; 2-factor model: experienced customer mistreatment + observed customer mistreatment; self-blame + shame + psychological withdrawal behavior + customer-directed blame + hostility + service sabotage.

**Table 2:** Summary statistics, zero-order correlations, and reliability coefficients.

Variable	1	2	3	4	5	6	7	8	9	10	11	12	13
1. Gender	—												
2. Age	−0.06	—											
3. Education	0.06	0.21 **	—										
4. Tenure	−0.10	0.44 **	−0.08	—									
5. Social desirability bias	−0.11	0.01	0.06	0.00	(0.72)								
6. Experienced customer mistreatment	−0.01	0.01	−0.06	−0.05	−0.18 **	(0.89)							
7. Observed customer mistreatment	0.00	−0.04	−0.07	0.00	−0.10	0.60 **	(0.90)						
8. Self-blame	−0.02	−0.02	0.06	−0.04	−0.10	0.05	−0.08	(0.87)					
9. Customer-directed blame	0.01	−0.04	−0.06	−0.07	0.06	0.47 **	0.28 **	0.12 *	(0.88)				
10. Shame	0.14 *	−0.09	−0.01	−0.10	−0.17 **	0.22 **	−0.02	0.38 **	0.22 **	(0.95)			
11. Hostility	0.02	0.02	−0.03	0.01	−0.01	0.34 **	0.22 **	−0.07	0.28 **	0.14 *	(0.94)		
12. Psychological withdrawal behaviors	0.07	−0.05	0.10	0.04	−0.23 **	0.13 *	0.01	0.26 **	0.10	0.33 **	0.11	(0.95)	
13. Service sabotage	0.03	−0.04	−0.02	0.06	−0.18 **	0.34 **	0.14 *	0.00	0.29 **	0.09	0.42 **	0.29 **	(0.94)
Mean	0.46	2.14	2.44	2.18	3.78	2.87	3.08	3.65	3.63	3.56	3.32	3.43	3.10
Std. Deviation	0.50	1.05	0.95	1.29	1.37	1.29	1.25	1.27	1.40	1.43	1.32	1.19	1.39

Note. *N* = 282; ** *p* < 0.01; * *p* < 0.05.

**Table 3:** (**a**) PROCESS results (Model 83-unstandardized coefficients) for the overall model. (**b**) PROCESS results (Model 83-unstandardized coefficients) for the overall model.

**(a)**									
**Variables**	**Self-Blame**			**Shame**			**Psychological Withdrawal** **Behaviors**		
	**Estimate (SE)**	**LLCI**	**ULCI**	**Estimate (SE)**	**LLCI**	**ULCI**	**Estimate (SE)**	**LLCI**	**ULCI**
Constant	4.10 ** (0.32)	3.456	4.736	2.55 ** (0.42)	1.726	3.383	2.37 ** (0.38)	1.616	3.118
Predictor variable									
Experienced customer mistreatment	0.28 ** (0.08)	0.136	0.431	0.21 ** (0.06)	0.089	0.325	0.05 (0.05)	−0.049	0.157
Moderator									
Observed customer mistreatment	−0.20 ** (0.07)	−0.339	−0.057						
Interaction									
Experienced customer mistreatment × Observed customer mistreatment	−0.20 ** (0.04)	−0.271	−0.131						
Mediator 1									
Self-blame				0.41 ** (0.06)	0.287	0.526	0.14 * (0.06)	0.030	0.250
Mediator 2									
Shame							0.19 ** (0.05)	0.089	0.291
Control variables									
Gender	−0.12 (0.14)	−0.407	0.163	0.40 * (0.16)	0.090	0.701	0.04 (0.13)	−0.222	0.303
Age	−0.01 (0.08)	−0.164	0.145	−0.08 (0.08)	−0.250	0.081	−0.12 (0.07)	−0.259	0.023
Education	0.08 (0.08)	−0.077	0.234	−0.02 (0.08)	−0.188	0.146	0.17 * (0.07)	0.026	0.309
Tenure	−0.05 (0.06)	−0.173	0.075	−0.04 (0.07)	−0.168	0.097	0.12 * (0.06)	0.003	0.228
Social desirability bias	−0.07 (0.05)	−0.174	0.037	−0.09 (0.06)	−0.206	0.020	−0.15 ** (0.05)	−0.242	−0.050
	R2 = 0.14			R2 = 0.22			R2 = 0.18		
	F (8, 273) = 5.47 *p* < 0.01			F (7, 274) = 11.11 *p* < 0.01			F (8, 273) = 7.67 *p* < 0.01		
**(b)**									
**Variables**	**Customer-Directed Blame**			**Hostility**			**Service Sabotage**		
	**Estimate (SE)**	**LLCI**	**ULCI**	**Estimate (SE)**	**LLCI**	**ULCI**	**Estimate (SE)**	**LLCI**	**ULCI**
Constant	3.17 ** (0.32)	2.533	3.797	2.60 ** (0.39)	1.827	3.373	1.85 ** (0.41)	1.046	2.659
Predictor variable									
Experienced customer mistreatment	0.41 ** (0.07)	0.262	0.553	0.29 ** (0.07)	0.154	0.417	0.16 * (0.07)	0.025	0.288
Moderator									
Observed customer mistreatment	0.01 (0.07)	−0.134	0.144						
Interaction									
Experienced customer mistreatment × Observed customer mistreatment	0.17 ** (0.04)	0.105	0.243						
Mediator 1									
Customer-directed blame				0.14 * (0.06)	0.016	0.256	0.14 * (0.06)	0.023	0.258
Mediator 2									
Hostility							0.35 ** (0.06)	0.232	0.462
Control variables									
Gender	0.11 (0.14)	−0.174	0.389	0.08 (0.15)	−0.220	0.373	0.02 (0.15)	−0.272	0.303
Age	−0.06 (0.08)	−0.208	0.098	0.03 (0.08)	−0.134	0.187	−0.15 (0.08)	−0.302	0.009
Education	−0.04 (0.08)	−0.195	0.112	−0.02 (0.08)	−0.181	0.142	0.08 (0.08)	−0.077	0.237
Tenure	−0.01 (0.06)	−0.136	0.109	0.02 (0.07)	−0.106	0.152	0.14 * (0.06)	0.013	0.262
Social desirability bias	0.13 * (0.05)	0.026	0.234	0.04 (0.06)	−0.074	0.147	−0.16 ** (0.05)	−0.266	−0.052
	R2 = 0.31			R2 = 0.14			R2 = 0.27		
	F (8, 273) = 15.09 *p* < 0.01			F (7, 274) = 6.17 *p* < 0.01			F (8, 273) = 12.48 *p* < 0.01		

Note. *N* = 282; ** *p* < 0.01; * *p* < 0.05; LLCI = lower limit of confidence interval; ULCI = upper limit of confidence interval.

**Table 4:** (**a**) Conditional and unconditional indirect effects of experienced customer mistreatment on psychological withdrawal behaviors. (**b**) Conditional and unconditional indirect effects of experienced customer mistreatment on service sabotage.

**(a)**								
**Levels of Observed Customer Mistreatment**	**Indirect Effect**	**Boot SE**	**Boot 95% CI**		**Index**	**Boot SE**	**Boot 95% CI**	
			**LLCI**	**ULCI**			**LLCI**	**ULCI**
	Experienced customer mistreatment → Self-blame → Shame → Psychological withdrawal behaviors							
−1SD (−1.25)	0.041	0.02	0.014	0.077	−0.016	0.01	−0.027	−0.005
M (0.00)	0.022	0.01	0.006	0.044				
+1SD (+1.25)	0.003	0.01	−0.010	0.016				
	Experienced customer mistreatment → Self−blame → Psychological withdrawal behaviors							
−1SD (−1.25)	0.075	0.04	0.004	0.170	−0.028	0.02	−0.063	−0.001
M (0.00)	0.040	0.02	0.002	0.096				
+1SD (+1.25)	0.004	0.01	−0.019	0.033				
	Experienced customer mistreatment → Shame → Psychological withdrawal behaviors							
—	0.039	0.02	0.008	0.087	—	—	—	—
**(b)**								
**Levels of Observed Customer Mistreatment**	**Indirect Effect**	**Boot SE**	**Boot 95% CI**		**Index**	**Boot SE**	**Boot 95% CI**	
			**LLCI**	**ULCI**			**LLCI**	**ULCI**
	Experienced customer mistreatment → Customer-directed blame → Hostility → Service sabotage							
−1SD (−1.25)	0.009	0.01	−0.001	0.028	0.008	0.00	−0.0004	0.0177
M(0.00)	0.019	0.01	−0.001	0.045				
+1SD (+1.25)	0.029	0.02	−0.001	0.064				
	Experienced customer mistreatment → Customer-directed blame → Service sabotage							
−1SD (−1.25)	0.027	0.02	−0.004	0.071	0.025	0.01	0.005	0.051
M (0.00)	0.057	0.03	0.011	0.114				
+1SD (+1.25)	0.088	0.04	0.019	0.170				
	Experienced customer mistreatment → Hostility → Service sabotage							
—	0.099	0.03	0.042	0.161	—	—	—	—

Note. *N* = 282; Bootstrap sample size = 10,000. LLCI = lower limit of confidence interval; ULCI = upper limit of confidence interval.

## Data Availability

The data that support the findings of this study are available from the corresponding authors upon reasonable request due to privacy.

## Abbreviations

The following abbreviations are used in this manuscript:

CMB	common method bias
SSGS	State Shame and Guilt Scale
PANAS-X	Positive and Negative Affect Schedule, Expanded Form
BIDR	Balanced Inventory of Desirable Responding
CFAs	confirmatory factor analyses
ML	maximum-likelihood

## References

[B1-behavsci-16-01385] Aiken L. S., West S. G., Reno R. R. (1991). Multiple regression: Testing and interpreting interactions.

[B2-behavsci-16-01385] Alola U. V., Olugbade O. A., Avci T., Öztüren A. (2019). Customer incivility and employees’ outcomes in the hotel: Testing the mediating role of emotional exhaustion. Tourism Management Perspectives.

[B3-behavsci-16-01385] Amarnani R. K., Restubog S. L. D., Shao R., Cheng D. C., Bordia P. (2022). A self-verification perspective on customer mistreatment and customer-directed organizational citizenship behaviors. Journal of Organizational Behavior.

[B4-behavsci-16-01385] Andiappan M., Treviño L. K. (2010). Beyond righting the wrong: Supervisor-subordinate reconciliation after an injustice. Human Relations.

[B5-behavsci-16-01385] Aquino K., Tripp T. M., Bies R. J. (2001). How employees respond to personal offense: The effects of blame attribution, victim status, and offender status on revenge and reconciliation in the workplace. Journal of Applied Psychology.

[B6-behavsci-16-01385] Baranik L. E., Wang M., Gong Y., Shi J. (2017). Customer mistreatment, employee health, and job performance: Cognitive rumination and social sharing as mediating mechanisms. Journal of Management.

[B7-behavsci-16-01385] Bedi A., Schat A. C. H. (2017). Employee revenge against uncivil customers. Journal of Services Marketing.

[B8-behavsci-16-01385] Berry C. M., Lelchook A. M., Clark M. A. (2012). A meta-analysis of the interrelationships between employee lateness, absenteeism, and turnover: Implications for models of withdrawal behavior. Journal of Organizational Behavior.

[B9-behavsci-16-01385] Bhuptani P. H., Messman T. L. (2023). Role of blame and rape-related shame in distress among rape victims. Psychological Trauma: Theory, Research, Practice, and Policy.

[B10-behavsci-16-01385] Blau G. J. (1985). Relationship of extrinsic, intrinsic, and demographic predictors to various types of withdrawal behaviors. Journal of Applied Psychology.

[B11-behavsci-16-01385] Bradfield M., Aquino K. (1999). The effects of blame attributions and offender likableness on forgiveness and revenge in the workplace. Journal of Management.

[B12-behavsci-16-01385] Brislin R. W., Triandis H. C., Berry J. W. (1980). Translation and content analysis of oral and written material. Handbook of cross-cultural psychology: Methodology.

[B13-behavsci-16-01385] Burton J. P., Taylor S. G., Barber L. K. (2014). Understanding internal, external, and relational attributions for abusive supervision. Journal of Organizational Behavior.

[B14-behavsci-16-01385] Cascardi M., O’Leary K. D. (1992). Depressive symptomatology, self-esteem, and self-blame in battered women. Journal of Family Violence.

[B15-behavsci-16-01385] Cheng J., Jiang J. (2026). Customer mistreatment and venting to conversational AI: Emotional exhaustion as mediator and trust in conversational AI as moderator. Behavioral Sciences.

[B16-behavsci-16-01385] Chi N. W., Chang H. T., Huang H. L. (2015). Can personality traits and daily positive mood buffer the harmful effects of daily negative mood on task performance and service sabotage? A self-control perspective. Organizational Behavior and Human Decision Processes 131.

[B17-behavsci-16-01385] Chi N. W., Tsai W. C., Tseng S.-M. (2013). Customer negative events and employee service sabotage: The roles of employee hostility, personality and group affective tone. Work & Stress.

[B18-behavsci-16-01385] Chi N. W., Yang J., Lin C. Y. (2018). Service workers’ chain reactions to daily customer mistreatment: Behavioral linkages, mechanisms, and boundary conditions. Journal Occupational Health Psychology.

[B19-behavsci-16-01385] Daniels M. A., Robinson S. L. (2019). The shame of it all: A review of shame in organizational life. Journal of Management.

[B20-behavsci-16-01385] Dawson J. F. (2014). Moderation in management research: What, why, when, and how. Journal of Business & Psychology.

[B21-behavsci-16-01385] Dhanani L. Y., LaPalme M. L. (2019). It’s not personal: A review and theoretical integration of research on vicarious workplace mistreatment. Journal of Management.

[B22-behavsci-16-01385] Fan L., Ma J., Fan Y., Yang Y. (2026). Beyond “tit-for-tat”: Understanding employees’ divergent reactions to customer mistreatment. International Journal of Conflict Management.

[B23-behavsci-16-01385] Fan L., Yang Y., Fan A., Ma J., Chen L. (2025). “Why you?!”: An attribution perspective on third-party employees’ unfavorable reactions to observed customer mistreatment. International Journal of Hospitality Management.

[B24-behavsci-16-01385] Fan L., Zhou X., Ren J., Ma J., Yang Y., Shao W. (2022). A self-regulatory perspective on the link between customer mistreatment and employees’ displaced workplace deviance: The buffering role of mindfulness. International Journal of Contemporary Hospitality Management.

[B25-behavsci-16-01385] Fetchenhauer D., Jacobs G., Belschak F. (2005). Belief in a just world, causal attributions, and adjustment to sexual violence. Social Justice Research.

[B26-behavsci-16-01385] Flanagan J. C. (1954). The critical incident technique. Psychological Bulletin.

[B27-behavsci-16-01385] Garcia P. R. J. M., Restubog S. L. D., Lu V. N., Amarnani R. K., Wang L., Capezio A. (2019). Attributions of blame for customer mistreatment: Implications for employees’ service performance and customers’ negative word of mouth. Journal of Vocational Behavior 110.

[B28-behavsci-16-01385] Garnefski N., Kraaij V., Spinhoven P. (2001). Negative life events, cognitive emotion regulation and emotional problems. Personality & Individual Differences.

[B29-behavsci-16-01385] Goetzke B., Nitzko S., Spiller A. (2014). Consumption of organic and functional food. A matter of well-being and health?. Appetite.

[B30-behavsci-16-01385] Grandey A. A., Dickter D. N., Sin H. P. (2004). The customer is not always right: Customer aggression and emotion regulation of service employees. Journal of Organizational Behavior.

[B31-behavsci-16-01385] Greenbaum R. L., Bonner J. M., Gray T. W., Mawritz M. B. (2020). Moral emotions: A review and research agenda for management scholarship. Journal of Organizational Behavior.

[B32-behavsci-16-01385] Greenbaum R. L., Mawritz M. B., Eissa G. (2012). Bottom-line mentality as an antecedent of social undermining and the moderating roles of core self-evaluations and conscientiousness. Journal of Applied Psychology.

[B33-behavsci-16-01385] Greenbaum R. L., Quade M. J., Mawritz M. B., Kim J., Crosby D. (2014). When the customer is unethical: The explanatory role of employee emotional exhaustion onto work-family conflict, relationship conflict with coworkers, and job neglect. Journal of Applied Psychology.

[B34-behavsci-16-01385] Harvey P., Madison K., Martinko M., Crook T. R., Crook T. A. (2014). Attribution theory in the organizational sciences: The road traveled and the path ahead. Academy of Management Perspectives.

[B35-behavsci-16-01385] Hayes A. F. (2018). Introduction to mediation, moderation, and conditional process analysis: A regression-based approach.

[B36-behavsci-16-01385] Hershcovis M. S., Cameron A.-F., Gervais L., Bozeman J. (2018). The effects of confrontation and avoidance coping in response to workplace incivility. Journal of Occupational Health Psychology.

[B37-behavsci-16-01385] Hershcovis M. S., Ogunfowora B., Reich T. C., Christie A. M. (2017). Targeted workplace incivility: The roles of belongingness, embarrassment, and power. Journal of Organizational Behavior.

[B38-behavsci-16-01385] Ho V. T., Gupta N. (2014). Retaliating against customer interpersonal injustice in a Singaporean context: Moderating roles of self-efficacy and social support. Applied Psychology.

[B39-behavsci-16-01385] Hobfoll (1989). Conservation of resources: A new attempt at conceptualizing stress. American Psychologist.

[B40-behavsci-16-01385] Huilian Z., Waqas M., Yahya F., Ahmad Qadri U., Zahid F. (2022). I have had enough: When and how customer mistreatment leads to coworker undermining. Frontiers in Psychology.

[B41-behavsci-16-01385] Izard C. E., Ackerman B. P., Schultz D., Singer J. A., Singer P. (1999). Independent emotions and consciousness: Self-consciousness and dependent emotions. At play in the fields of consciousness: Essays in honor of Jerome L. Singer.

[B42-behavsci-16-01385] Jalil D., Xu X., Jiang L., Wang H.-J. (2022). Do not ask, but you shall still receive: Newcomer reactions to receiving negative gossip. Stress and Health.

[B43-behavsci-16-01385] Janoff-Bulman R. (1979). Characterological versus behavioral self-blame: Inquiries into depression and rape. Journal of Personality & Social Psychology.

[B44-behavsci-16-01385] Kahneman D., Miller D. T. (1986). Norm theory: Comparing reality to its alternatives. Psychological Review.

[B45-behavsci-16-01385] Kedharnath U., Henle C. A., Mumford T. (2024). Attributions for abusive supervision: Who do subordinates blame and does it matter?. Scandinavian Journal of Psychology.

[B46-behavsci-16-01385] Kelley H. H. (1973). The processes of causal attribution. American Psychologist.

[B47-behavsci-16-01385] Kelley H. H., Michela J. L. (1980). Attribution theory and research. Annual review of psychology.

[B48-behavsci-16-01385] Khademi-Gerashi M., Akhgari F., Damberg S., Moradi F. (2024). Pandemic-oriented customer mistreatment, service sabotage and service performance: A self-presentation perspective. International Hospitality Review.

[B49-behavsci-16-01385] Kim H., Qu H. (2018). The effects of experienced customer incivility on employees’ behavior toward customers and coworkers. Journal of Hospitality & Tourism Research.

[B50-behavsci-16-01385] Kim H., Qu H. (2019). Employees’ burnout and emotional intelligence as mediator and moderator in the negative spiral of incivility. International Journal of Contemporary Hospitality Management.

[B51-behavsci-16-01385] Lehman W. E., Simpson D. D. (1992). Employee substance use and on-the-job behaviors. Journal of Applied Psychology.

[B52-behavsci-16-01385] Leng L., Beckers T., Vervliet B. (2024). What a relief! The pleasure of threat avoidance. Emotion.

[B53-behavsci-16-01385] Lerner M. J., Miller D. T. (1978). Just world research and the attribution process: Looking back and ahead. Psychological Bulletin.

[B54-behavsci-16-01385] Lerner M. J., Simmons C. H. (1966). Observer’s reaction to the” innocent victim”: Compassion or rejection?. Journal of Personality & Social Psychology.

[B55-behavsci-16-01385] Li S., Zhan J., Cheng B., Scott N. (2021). Frontline employee anger in response to customer incivility: Antecedents and consequences. International Journal of Hospitality Management.

[B56-behavsci-16-01385] Liu D., Zhou C., Wu Y. (2024). The self-distancing perspective of daily customer mistreatment and employee service behaviors. Journal of Hospitality and Tourism Management 61.

[B57-behavsci-16-01385] MacKinnon D. P., Lockwood C. M., Williams J. (2004). Confidence limits for the indirect effect: Distribution of the product and resampling methods. Multivariate Behavioral Research.

[B58-behavsci-16-01385] Marr J. C., Lemay E. P., Park H. (2025). Conflicted about coworkers: How coworker support influences engagement after status loss. Personnel Psychology.

[B59-behavsci-16-01385] Marschall D., Sanftner J., Tangney J. P. (1994). The state shame and guilt scale.

[B60-behavsci-16-01385] Medea A., Thompson K. (1974). Against rape.

[B61-behavsci-16-01385] Miller D. T., Porter C. A. (1983). Self-blame in victims of violence. Journal of Social Issues.

[B62-behavsci-16-01385] Miranda G. A., Welbourne J. L., Sariol A. M. (2020). Feeling shame and guilt when observing workplace incivility: Elicitors and behavioral responses. Human Resource Development Quarterly.

[B63-behavsci-16-01385] Mitchell M. S., Ambrose M. L. (2012). Employees’ behavioral reactions to supervisor aggression: An examination of individual and situational factors. Journal of Applied Psychology.

[B64-behavsci-16-01385] Mitchell M. S., Vogel R. M., Folger R. (2015). Third parties’ reactions to the abusive supervision of coworkers. Journal of Applied Psychology.

[B65-behavsci-16-01385] Mussweiler T., Rüter K., Epstude K. (2004). The man who wasn’t there: Subliminal social comparison standards influence self-evaluation. Journal of Experimental Social Psychology.

[B66-behavsci-16-01385] Muthén L. K., Muthén B. O. (1998–2012). Mplus user’s guide. Los angeles.

[B67-behavsci-16-01385] Ni D., Liu X., Zheng X. (2024). Render good for evil? The relationship between customer mistreatment and customer-oriented citizenship behavior. Journal of Business Research.

[B68-behavsci-16-01385] Niedenthal P. M., Tangney J. P., Gavanski I. (1994). If only I weren’t” versus “if only I hadn’t”: Distinguishing shame and guilt in conterfactual thinking. Journal of Personality & Social Psychology.

[B69-behavsci-16-01385] Pan W., Sun L. Y. (2023). Do victims really help their abusive supervisors? Reevaluating the positive consequences of abusive supervision. Behavioral Sciences.

[B70-behavsci-16-01385] Park J., Kim H. J. (2020). Customer mistreatment and service performance: A self-consistency perspective. International Journal of Hospitality Management.

[B71-behavsci-16-01385] Paustian-Underdahl S. C., Devine R. A., Hideg I., Holmes R. M., Lamont B. T., Lam J. Y. (2025). Mind the gap: A psychological and structural perspective on activist shareholders’ targeting of women CEOs. Journal of Management.

[B72-behavsci-16-01385] Peng A. C., Schaubroeck J. M., Li Y. (2014). Social exchange implications of own and coworkers’ experiences of supervisory abuse. Academy of Management Journal.

[B73-behavsci-16-01385] Podsakoff P. M., MacKenzie S. B., Lee J. Y., Podsakoff N. P. (2003). Common method biases in behavioral research: A critical review of the literature and recommended remedies. Journal of Applied Psychology.

[B74-behavsci-16-01385] Podsakoff P. M., MacKenzie S. B., Podsakoff N. P. (2012). Sources of method bias in social science research and recommendations on how to control it. Annual Review Psychology.

[B75-behavsci-16-01385] Rancher C., McDonald R., Kamata A., Jackson M., Jouriles E. N. (2022). Self-blame in adolescents who have been sexually abused: Factor structure and differential correlates of abuse-specific and global measures. Assessment.

[B76-behavsci-16-01385] Ruebottom T., Toubiana M. (2024). Embodied shame and organization studies. Organization Studies.

[B77-behavsci-16-01385] Schilpzand P., Leavitt K., Lim S. (2016). Incivility hates company: Shared incivility attenuates rumination, stress, and psychological withdrawal by reducing self-blame. Organizational Behavior and Human Decision Processes.

[B78-behavsci-16-01385] Shao P., Li A., Mawritz M. (2018). Self-protective reactions to peer abusive supervision: The moderating role of prevention focus and the mediating role of performance instrumentality. Journal of Organizational Behavior.

[B79-behavsci-16-01385] Shao R., Skarlicki D. P. (2014). Service employees’ reactions to mistreatment by customers: A comparison between north America and east Asia. Personnel Psychology.

[B80-behavsci-16-01385] Shaver K. G. (1970). Defensive attribution: Effects of severity and relevance on the responsibility assigned for an accident. Journal of Personality & Social Psychology.

[B81-behavsci-16-01385] Skarlicki D. P., Ellard J. H., Kelln B. R. (1998). Third-party perceptions of a layoff: Procedural, derogation, and retributive aspects of justice. Journal of Applied Psychology.

[B82-behavsci-16-01385] Skarlicki D. P., Kulik C. T. (2005). Third-party reactions to employee (Mis)treatment: A justice perspective. Research in Organizational Behavior.

[B83-behavsci-16-01385] Skarlicki D. P., Turner R. A. (2014). Unfairness begets unfairness: Victim derogation bias in employee ratings. Organizational Behavior and Human Decision Processes.

[B84-behavsci-16-01385] Skarlicki D. P., van Jaarsveld D. D., Walker D. D. (2008). Getting even for customer mistreatment: The role of moral identity in the relationship between customer interpersonal injustice and employee sabotage. Journal of Applied Psychology.

[B85-behavsci-16-01385] Sliter M., Sliter K., Jex S. (2012). The employee as a punching bag: The effect of multiple sources of incivility on employee withdrawal behavior and sales performance. Journal of Organizational Behavior.

[B86-behavsci-16-01385] Taylor S. E., Lichtman R. R., Wood J. V. (1984). Attributions, beliefs about control, and adjustment to breast cancer. Journal of Personality & Social Psychology.

[B87-behavsci-16-01385] Tennen H., Affleck G., Gershman K. (1986). Self-blame among parents of infants with perinatal complications: The role of self-protective motives. Journal of Personality & Social Psychology.

[B88-behavsci-16-01385] Tong J., Chong S., Johnson R. E. (2019). The indirect relations of workplace incivility with emotional exhaustion and supportive behaviors via self-blame: The moderating roles of observed incivility and trait emotional control. Journal of Organizational Behavior.

[B89-behavsci-16-01385] Tracy J. L., Robins R. W. (2004). Putting the self into self-conscious emotions: A theoretical model. Psychological Inquiry.

[B90-behavsci-16-01385] Tröster C., Van Quaquebeke N. (2021). When victims help their abusive supervisors: The role of LMX, self-blame, and guilt. Academy of Management Journal.

[B91-behavsci-16-01385] Walker D. D., van Jaarsveld D. D., Skarlicki D. P. (2014). Exploring the effects of individual customer incivility encounters on employee incivility: The moderating roles of entity (in)civility and negative affectivity. Journal of Applied Psychology.

[B92-behavsci-16-01385] Wang I. A., Chen P. C., Chi N. W. (2023). Mitigating immediate and lagged effects of customer mistreatment on service failure and sabotage: Critical roles of service recovery behaviors. Journal of Business Research.

[B93-behavsci-16-01385] Wang M., Liao H., Zhan Y., Shi J. (2011). Daily customer mistreatment and employee sabotage against customers: Examining emotion and resource perspectives. Academy of Management Journal.

[B94-behavsci-16-01385] Wang M., Liu S., Liao H., Gong Y., Kammeyer-Mueller J., Shi J. (2013). Can’t get it out of my mind: Employee rumination after customer mistreatment and negative mood in the next morning. Journal of Applied Psychology.

[B95-behavsci-16-01385] Wang X., Wang H. (2017). How to survive mistreatment by customers: Employees’ work withdrawal and their coping resources. International Journal of Conflict Management.

[B96-behavsci-16-01385] Wang Z., Jiang F. (2023). It is not only what you do, but why you do it: The role of attribution in employees’ emotional and behavioral responses to illegitimate tasks. Journal of Vocational Behavior.

[B97-behavsci-16-01385] Watson D., Clark L. A. (1994). The panas-X: Manual for the positive and negative affect schedule-expanded form *[Unpublished manuscript]*.

[B98-behavsci-16-01385] Weiner B. (1986). Principles for a theory of motivation. An attributional theory of motivation and emotion. SSSP springer series in social psychology.

[B99-behavsci-16-01385] Winkler N., Kroh M., Spiess M. (2006). Entwicklung einer deutschen kurzskala zur zweidimensionalen messung von sozialer erwünschtheit [Development of a German short scale for two-dimensional measurement of social desirability].

[B100-behavsci-16-01385] Wong J. W. C., Pan S. Y. (2023). Different emotional and behavioral reactions to customer mistreatment among hotel employees: A multilevel moderated mediation model. Journal of Hospitality and Tourism Management.

[B101-behavsci-16-01385] Wu Y., Groth M., Zhang K., Minbashian A. (2023). A meta-analysis of the impact of customer mistreatment on service employees’ affective, attitudinal and behavioral outcomes. Journal of Service Management.

[B102-behavsci-16-01385] Yang F., Lu M., Huang X. (2020). Customer mistreatment and employee well-being: A daily diary study of recovery mechanisms for frontline restaurant employees in a hotel. International Journal of Hospitality Management.

[B103-behavsci-16-01385] Zhan Y., Wang M., Shi J. (2016). Interpersonal process of emotional labor: The role of negative and positive customer treatment. Personnel Psychology.

[B104-behavsci-16-01385] Zhang H., Zhou Z. E., Zhang L., Liu Y., Shi Y. (2024). How customer mistreatment hinders employee sleep quality and next-morning vigor: The effects of affective rumination and mindfulness. Applied Psychology.

[B105-behavsci-16-01385] Zhang R., Wu Y., Ferreira-Meyers K. (2019). The work-family spillover effects of customer mistreatment for service employees: The moderating roles of psychological detachment and leader–member exchange. Frontiers in Psychology.

[B106-behavsci-16-01385] Zhu H., Lyu Y., Ye Y. (2021). The impact of customer incivility on employees’ family undermining: A conservation of resources perspective. Asia Pacific Journal of Management.

